# Investigation of Removing Basic Yellow 28 and Basic Blue 3 Dyes from Water Using Mulberry Leaves (*Morus nigra* L.) and Assessment of Ultrasonic Effects

**DOI:** 10.3390/molecules30173539

**Published:** 2025-08-29

**Authors:** Adella Myori Hardieka, Türkan Börklü Budak

**Affiliations:** Department of Chemistry, Faculty of Art and Science, Yıldız Technical University, 34220 İstanbul, Türkiye; adella.hardieka@std.yildiz.edu.tr

**Keywords:** adsorption, Basic Yellow 28, Basic Blue 3, mulberry leaves, wastewater, ultrasonic bath-assisted

## Abstract

Many industries release untreated synthetic dye effluents into water bodies, harming ecosystems and human health. Therefore, an economical and sustainable solution for treating dye-contaminated water must be developed. In this study, mulberry leaves (*Morus nigra* L.), as a cost-effective and sustainable adsorbent, were prepared to remove Basic Yellow 28 (BY28) and Basic Blue 3 (BB3) cationic dyes from industrial dye wastewater using adsorption. Batch experiments with key variables such as initial dye concentration, adsorbent dosage, contact time, temperature, stirring speed, and pH were conducted to find optimal conditions. The effectiveness of mulberry leaves as an adsorbent after multiple regeneration cycles was examined. The adsorbent was characterized through various instrumental methods, including FTIR, SEM, XRD, and BET analysis. Adsorption performance was analyzed using the Langmuir and Freundlich isotherm models. The results showed that the mulberry leaf adsorbent best fits the Langmuir model, with R^2^ values of 0.999 for BY28 and 0.973 for BB3. The maximum adsorption capacities were 0.15 mg/g for BY28 and 7.19 mg/g for BB3, indicating their upper limits for dye uptake. The optimal conditions achieving removal efficiencies of over 99% were 1.5 g, 50 mL, 15 min, 180 rpm, and 10 mg/L at 30 °C for BY28 in neutral pH (7) and 1.5 g, 50 mL, 45 min, 100 rpm, and 30 mg/L at 40 °C for BB3 in basic pH (10). The regeneration of mulberry leaves as an adsorbent through acid treatment with 0.1 M HCl and 0.1 M CH_3_COOH solutions maintained a high performance, achieving up to 98% dye removal efficiency after two regeneration cycles. It has been observed that successful results can be achieved in terms of reusability. Additionally, the removals of BB3 and BY28 performed in an ultrasonic-bath-assisted environment successfully achieved removal efficiencies of 84.87% and 75.41%, respectively. According to the results, mulberry leaves can effectively be used in wastewater treatment to remove dyes, can be reused multiple times, and thus serve as an environmentally friendly and sustainable adsorbent.

## 1. Introduction

Water represents an irreplaceable natural resource fundamental to all biological life. The survival of humans, flora, and fauna is fundamentally dependent on water availability, making its quality paramount for both ecosystem integrity and public health [1]. The accumulation of vast amounts of organic and inorganic pollutants in water bodies, including rivers, oceans, lakes, and streams, has become a global problem resulting from the massive discharge of industrial effluents and limited access to freshwater. Increasing population growth, industrialization, civilization, agricultural activities, domestic waste, and rapid urbanization are the major causes of water contamination [2,3]. Untreated or inadequately treated wastewater containing heavy metals, dyes, and toxic substances that flow into freshwater resources can negatively impact human health and the whole ecosystem, even at low concentrations [4,5,6].

Dyes are an essential parameter and are not only extensively applied in the textile industry but also in various other industries such as cosmetics, tanneries, leather, pulp mills, rubber, paper, plastics, pharmaceuticals, foods and beverages, and other industries [7]. Dyes are classified as chromophores and auxochromes based on their chemical structure. They can also be categorized depending on their solubility in water, like vat, sulfur, and dispersion dyes, which are insoluble in water. On the other hand, direct, reactive, azo, acidic, and basic dyes have high solubility in water [8]. Industrial effluents containing synthetic dyes exhibit significantly high chemical oxygen demand (COD) and biochemical oxygen demand (BOD) levels. In addition, due to their complex aromatic molecular structures, cationic and anionic dyes are non-biodegradable organic compounds. It also has a high potential to be carcinogenic and mutagenic [9,10]. Cationic dyes, including Basic Yellow 28 (BY28) and Basic Blue 3 (BB3), are the most commonly used in the textile industry. Both dyes can cause various diseases, such as mutagenicity, jaundice, respiratory problems, vomiting, skin allergies, cancer, and eye irritation [11].

Researchers are developing practical, easy-to-operate, eco-friendly wastewater treatments to eliminate synthetic dye-polluted water [12]. Several techniques exist to get rid of toxic dye contaminants from industrial wastewater, such as membrane filtration [13,14,15], photocatalysis degradation [16,17,18], ion exchange resin [19,20,21], coagulation [22,23], electrochemical treatment [24,25], biosorption [26,27], and adsorption [28,29,30,31]. These techniques may prove effective in removing toxic dyes from industrial wastewater. Despite this, some of them are technically costly and difficult to perform. The adsorption method has garnered significant attention as an advanced wastewater treatment method, particularly for synthetic dye effluents. Adsorption is the separation of solutes in a liquid based on the adsorbate–adsorbent interaction. This method is extensively used due to its cost-effectiveness, efficiency, the different kinds of adsorbents available, and the ease of regenerating adsorbents for reuse [32]. It can also remove both soluble and insoluble organic pollutants. According to some studies, with this method, percentage removal values can reach around 99.90% depending on the initial concentration [33,34].

Nowadays, several adsorbents have been studied by many scientists for the development of environmentally friendly, effective, and practical employments of adsorbents for dye degradation from industrial wastewater, including activated carbon [35,36], chitosan [37], alumina [38], kaolin [39], natural clays [40], zeolites [41], and agricultural waste or bio-waste [42,43,44,45]. Both farming and bio-waste are gaining popularity in environmental preservation due to their eco-friendly, biodegradable, economical, and renewable nature, making them extensively used as adsorbents for removing toxic dyes in industrial wastewater [46]. Mulberry leaves (*Morus nigra* L.) are one of the agricultural wastes that have shown potential as an adsorbent owing to their rich chemical composition, which includes cellulose, hemicellulose, and lignin, providing a surface for adsorbing numerous contaminants [47]. Previous studies have demonstrated that, as a natural adsorbent, mulberry leaves possess excellent adsorption capabilities for removing heavy metals [48,49], organic compounds, and other hazardous chemicals [50]. One advantage of using natural materials as adsorbents is that they can quickly regenerate. Regeneration can be performed through desorption, allowing the removed dye to be recovered and the adsorbent to be reused. The process is carried out by contacting the adsorbent with a solution known as a desorption agent, which can be acidic, basic, or neutral.

*Morus nigra*, commonly known as black mulberry (*Moraceae* family), belongs to the Morus genus, a native Asian tree, and is nowadays widely distributed in North Africa, Europe, and North and South America. It is a rapidly growing deciduous plant found in a broad range of climates and soil conditions worldwide [51]. It is also abundantly available in Türkiye and has gained a lot of attention for its diverse medicinal properties (including anti-diabetic effects [52,53], antioxidant [54,55] and immune-boosting effects [56]), and nutritional benefits [57,58]. The leaves of black mulberry (*Morus nigra* L.) are dark green and glossy, with a length of up to 20 cm. They are heart-shaped with a serrated edge and may seem lobed. Besides that, it has been proven successful as an adsorbent based on previous studies [59].

In this study, the removal of synthetic dyes BY28 and BB3 has been investigated by adsorption techniques using mulberry leaves (*Morus nigra* L.) as an adsorbent.

The effects of various key parameters on dye removal, including initial dye concentration, adsorbent amount, contact time, temperature, stirring speed, and pH, were investigated. Experiments were repeated with different water sources, maintaining the found optimum conditions. Again, experiments were conducted to evaluate the effects of ultrasonic baths, using the same optimum operating conditions. The sustainable management of used adsorbents, involving their regeneration for reuse with minimal degradation of adsorption capacity (q_e_), was also studied. The objective of the proposed study is to contribute to preventing water pollution. The proposed study investigated the removal of BY28 and BB3 dyes from aqueous media using mulberry leaves. This study aims to contribute to the literature and the field of wastewater treatment with an environmentally friendly, natural, and abundant material.

## 2. Experimental Procedure

### 2.1. Materials

The mulberry leaves (*Morus nigra* L.) used in this study were collected from İstanbul, Türkiye.

All adsorption experiments were conducted using analytical-grade chemicals without further purification. Both cationic dyes, Basic Yellow 28 (BY28; C_21_H_27_N_3_O_5_S, molecular weight = 433.5 g/mol, λ_max_ = 438 nm) and Basic Blue 3 (BB3; C_20_H_26_ClN_3_O, molecular weight = 359.89 g/mol, λ_max_ = 654 nm), were purchased from Sigma-Aldrich (Merck KGaA, St. Louis, MO, USA) and utilized as supplied. Stock solutions of 1.0 g/L of BY28 and BB3 dyes were prepared with distilled water, and then working solutions were obtained by dilution to the desired concentration. The influence of pH on dye adsorption was evaluated using buffered solutions at pH 4, 7, and 10. Citrate buffer solutions for pH 4, phosphate for pH 7, and carbonate for pH 10 were used, respectively. These solutions were purchased from Acros Organics (Thermo Fisher Scientific, Geel, Belgium). For adsorbent regeneration studies, 0.1 M solutions of HCl, NaOH, and CH_3_COOH were prepared. In contrast, a 0.1 M NaNO_3_ solution was employed for point-of-zero-charge (PZC) determination.

### 2.2. Instrumentation

The experimental procedures utilized the following instrumentation: an analytical balance (Radwag Balances and Scales, Radom, Poland), an oven (Memmert GmbH + Co. KG, Schwabach, Germany), a water purification system (Mikrotest MSD-08, Türkiye) to obtain distilled water, a UV-Vis spectrophotometer (Agilent 8453, Agilent Technologies, Santa Clara, CA, USA), a temperature-controlled shaking water bath (Julabo SW22), an ultrasonic water bath (ISOLAB Laborgeräte GmbH, Eschau, Germany), and a pH meter (WTW InoLab Cond pH 720, Germany). Fourier-transform infrared spectroscopy with attenuated total reflectance (FTIR-ATR; Nicolet IS10, Thermo Fisher Scientific Inc., Waltham, MA, USA) was employed for functional group analysis. Furthermore, comprehensive adsorbent material characterization was conducted, including morphological evaluation via scanning electron microscopy (SEM; Zeiss EVO LS10, Oberkochen, Germany), settings: 10 μm, EHT = 10.00 kV, WD = 8.5 mm, Signal A = SE1, Mag = 5.00 KX, Mag = 1.00 KX, and crystallographic analysis (phase composition degree and crystallite size), and by X-ray diffraction (XRD; Malvern PANalytical X’Pert PRO, UK), settings: 40 mA, 45 kV, goniometer radius [mm] 240.00, dist. focus-diverg. slit [mm] 144.50. The adsorbent’s textural properties (specific surface area, pore volume) were determined through nitrogen physisorption measurements using the Brunauer–Emmett–Teller (BET) method (Micromeritics ASAP 2020, Norcross, GA, USA), settings: Analysis adsorptive: N_2_, analysis bath temp.: −195.441 °C, equilibration interval: 10 s.

### 2.3. Preparation of the Adsorbent

**Figure 1:**
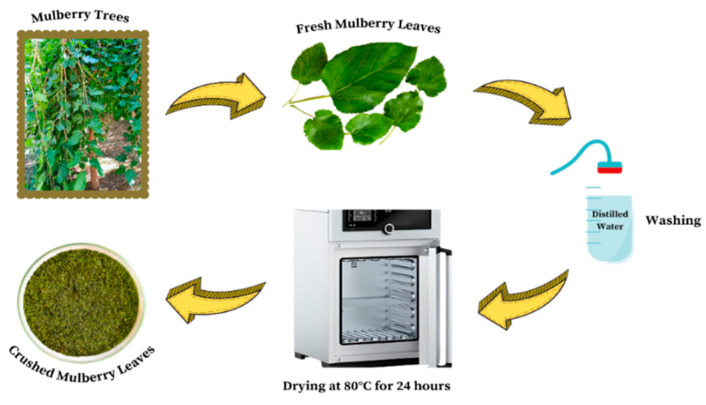
Preparation of mulberry leaf adsorbent.

The mulberry leaves were meticulously washed first with tap water and then with distilled water to remove any water-soluble impurities and foreign particles. The mulberry leaves were then subjected to a drying process, which took place at 80 °C for 24 h in an oven. Following this, the leaves were crushed into fine powder. Thereafter, adsorbent powders were sifted through a sieve with a defined particle size (60–80 mesh). The adsorbent was then stored in a dry, tightly sealed glass bottle. The preparation of the mulberry leaf adsorbent is illustrated in Figure 1.

### 2.4. Adsorption Process of BY28 and BB3

To investigate the adsorption of BY28 and BB3 dyes, 50 mL of BY28 and BB3 at specific concentrations from the stock solutions were separately transferred into 100 mL glass beakers. Batch adsorption studies were systematically performed under controlled laboratory conditions (25 °C) using a temperature-regulated water bath shaker. The influence of various parameters on the dye adsorption process, such as initial dye concentration (5–50 mg/L), adsorbent dosage (0.1–1.5 g), contact time (5–180 min), temperature (25–40 °C), agitation rate (100–180 rpm), and pH solution (4, 7, 10), has been investigated. By the end of the adsorption process, the sample solution had been filtered using filter paper (Whatman Cat. No. 1001, 150) and placed in test tubes for subsequent analysis. The filtered dye solutions were analyzed using a UV-Vis spectrophotometer to determine the absorbance value at λ_max_ (438 nm and 654 nm for BY28 and BB3, respectively) [60,61,62]. To determine the maximum wavelength of the dyes used, measurements were made between 200 and 750 nm using a UV spectrophotometer. In this case, wavelengths of 438 nm for BY28 and 654 nm for BB3 were determined by literature data. A measurement curve was then created between dye concentration and absorbance. The standard curve ranges were 5–25 mg/L for BY28 and 2–10 mg/L for BB3. Using the measurement curve obtained according to the Lambert–Beer law, absorbance values found in later stages of the study were converted to concentration, and % removal values were obtained using Equations (1) and (2). To calculate the removal efficiency (%R) and adsorption capacity (q_e_) of BY28 and BB3 dyes in solution, we used the following equations:

(1) $$\%R=\frac{{(C}_{o}-C_{e})}{C_{o}}\times 100$$

(2) $$\;\;\;q_{e}=\frac{{(C}_{o}-C_{e})}{m}\times V$$

where C_0_ denotes the initial dye concentration, C_e_ represents the equilibrium concentration of dye at time t (mg/L), m corresponds to the adsorbent dosage used (g), and V indicates the total volume of the dye solution (L).

### 2.5. Adsorption Isotherms

Adsorption isotherms represent a fundamental principle in designing and optimizing adsorption systems. These equilibrium models describe the quantitative relationship between adsorbate concentration (BY28 and BB3 dyes) and adsorption capacity per unit of adsorbent (mulberry leaves) at a constant temperature [63,64]. Various isotherm models provide critical insights into adsorption mechanisms, including the nature of adsorbate–adsorbent interactions and the maximum uptake capacity of the adsorption process. In this study, the Langmuir and Freundlich models, two widely applied isotherm models in adsorption studies, were utilized to identify the optimal fitting isotherm for both dyes. These isotherms depict the relationship between the adsorbed substance and the adsorbent material, with the Langmuir (Equations (3) and (4)) and Freundlich (Equation (5)) linear forms being the most frequently employed. The Langmuir isotherm characterizes monolayer adsorption, assuming the adsorbent surface consists of uniform sites. The Freundlich adsorption isotherms analyze reversible and heterogeneous adsorbent surfaces and explain multilayer adsorption on adsorbents with various functional groups, showing distinct adsorbent–adsorbate interactions [65]. The Langmuir and Freundlich isotherm models can be represented in linear form by the following equations:

(3) $$\frac{C_{e}}{q_{e}}=\frac{1}{K_{L}q_{max}}+\frac{C_{e}}{q_{max}}$$

(4) $$\frac{1}{q_{e}}=\frac{1}{K_{L}q_{max}}\times \frac{1}{C_{e}}+\frac{1}{q_{max}}$$

(5) $$Logq_{e}=LogK_{f}+\frac{1}{n}LogC_{e}$$

The non-linear analysis method is applied to eliminate errors caused by distinct estimations derived from the basic linear regression of the linearized versions of the Langmuir equations, which can considerably alter the R^2^ values. Non-linear analysis was employed as an effective method to prevent such problems. The Langmuir and Freundlich isotherm model can be expressed in their non-linear form as follows:

(6) $$q_{e}=\frac{q_{max}\times K_{L}\times C_{e}}{\left(1+K_{L}\times C_{e}\right)}$$

(7) $$q_{e}=K_{f}\times C_{e}^{\frac{1}{n}}$$

In Equations (3), (4) and (6), C_e_ represents the equilibrium dye concentration (mg/L), q_e_ denotes the adsorption capacity per unit mass of mulberry leaves (mg/g), q_max_ signifies the theoretical maximum adsorption capacity (mg/g), and K_L_ (L/mg) is the Langmuir constant, reflecting the binding affinity between the adsorbent and dye. Equations (5) and (7) incorporate the Freundlich coefficients K_f_ and n, which are related to the adsorption capacity and intensity, respectively. The adsorption process is favorable when the heterogeneity factor (1/n) is 0.1 ≤ 1/n ≤ 0.5, while 1/n values exceeding 2 indicate unfavorable adsorption conditions [66].

## 3. Results and Discussion

### 3.1. Characterization of Mulberry Leaf Adsorbent Before and After Adsorption

#### 3.1.1. SEM Analysis

**Figure 2:**
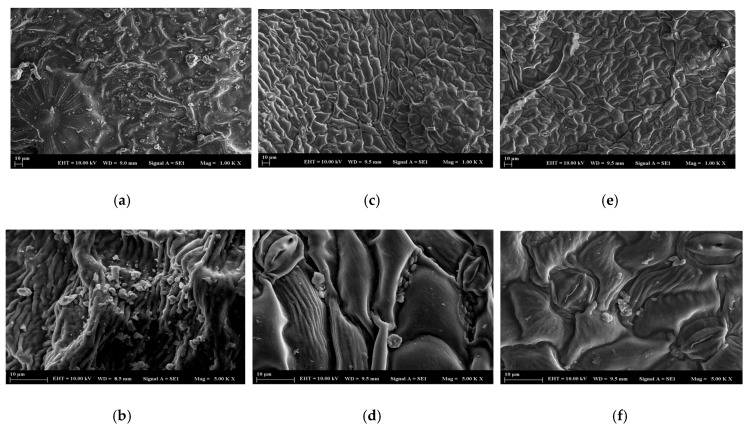
SEM images of natural mulberry leaves: (**a**,**b**) BY28-treated (**c**,**d**), and BB3-treated (**e**,**f**).

To determine the surface morphology of mulberry leaves before and after dye adsorption, measurements were carried out using a scanning electron microscope (SEM). Figure 2a,b show SEM images of mulberry leaves before adsorption (Figure 2a: 1.0 KX magnification range, EHT 10.0 kV, WD 9.0 mm, and particle size 10 µm; Figure 2b: 5.0 KX magnification range, EHT 10.0 kV, WD 8.5 mm, and particle size 10 µm). As can be seen here, the porous structure of mulberry leaves proves that they have the appropriate surface properties required for adsorption. After dye adsorption, the adsorbent properties of mulberry leaves were investigated under the same conditions, as shown in Figure 2c–f. The less rough surface seen in Figure 2c,d may be related to the adsorption of the BY28 dye on the adsorbent surface. Similarly, the images in Figure 2e,f can be interpreted as the presence of the BB3 dye on the adsorbent surface. 

#### 3.1.2. FTIR Analysis

FTIR spectra aid in identifying the functional groups involved in the adsorption mechanisms. A Fourier transform infrared-attenuated total reflectance spectroscopy (FTIR-ATR) analysis was conducted on the mulberry leaf adsorbent in both the pre- and post-adsorption processes by scanning the spectral range of 800–4000 cm^−1^.

**Figure 3:**
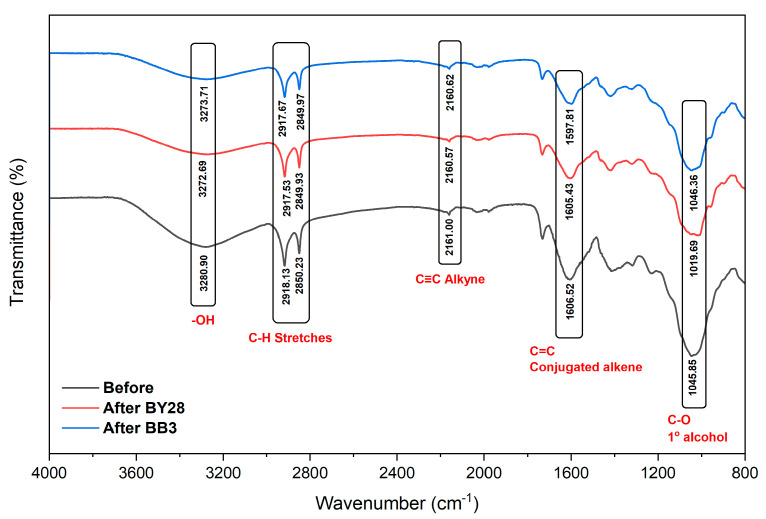
The FTIR-ATR spectra of mulberry crushed leaves powder before and after adsorption of BY28 and BB3 dyes.

The FTIR-ATR spectra of the mulberry leaf adsorbents before and after BY28 and BB3 dye adsorption are displayed in Figure 3. The FTIR spectral profiles exhibited comparable absorption bands pre- and post-adsorption, though variations in band intensities were observed. The peaks at 3280 cm^−1^, 2918 cm^−1^ to 2850 cm^−1^, and 2161 cm^−1^ were correlated to the O–H stretching vibration of hydroxyl groups, C–H stretching of carboxyl groups, and C≡C alkyne of the functional group carbon–carbon triple bonds, respectively [67]. The spectrum revealed two dominant peaks at 1606 cm^−1^ and 1045 cm^−1^, assigned to C=C stretching in conjugated alkene systems (or -CH_2_ scissoring vibrations) and C-O stretching of ether linkages, respectively [68].

Post-adsorption characterization of the mulberry leaf adsorbent’s functional groups was performed following exposure to BY28 and BB3 dyes under optimized experimental conditions. The FTIR spectra after BY28 dye adsorption exhibit distinct peaks at wave numbers ranging from 2917 to 2849 cm^−1^, indicating the presence of C–H stretching functional groups. A peak with a wave number of 3272 cm^−1^, the O–H group, was detected [69]. The spectral band at 2160 cm^−1^ was assigned to C≡C stretching vibrations characteristic of alkyne functional groups [70], while the peaks observed at wave numbers 1605 and 1019 cm^−1^ were related to the C=C conjugated alkene bond and C-O functional group, respectively [71]. Similarly, after BB3 dye adsorption, the broadband at wave number 3273 cm^−1^ was assigned to O–H stretching vibrations, indicative of hydroxyl groups. The spectral region between 2917 and 2849 cm^−1^ exhibited peaks characteristic of asymmetric C–H stretching vibrations, attributable to the aliphatic CH_2_ group [72]. A distinct band at 2160 cm^−1^ confirmed the presence of C≡C triple bond stretching (alkyne functional group). The peaks at 1597 cm^−1^ and 1046 cm^−1^ were identified as C=C conjugated alkene stretching and C–O bond vibrations, respectively.

FTIR-ATR spectral analysis before and after dye adsorption confirms the stability of functional groups and molecular vibrations, indicating that the adsorption mechanism is primarily surface-bound without significantly modifying the adsorbent’s chemical composition. This conclusion is further supported by SEM morphological characterization, which reveals no structural degradation or chemical transformation of the adsorbent surface post-adsorption.

#### 3.1.3. XRD Analysis

**Figure 4:**
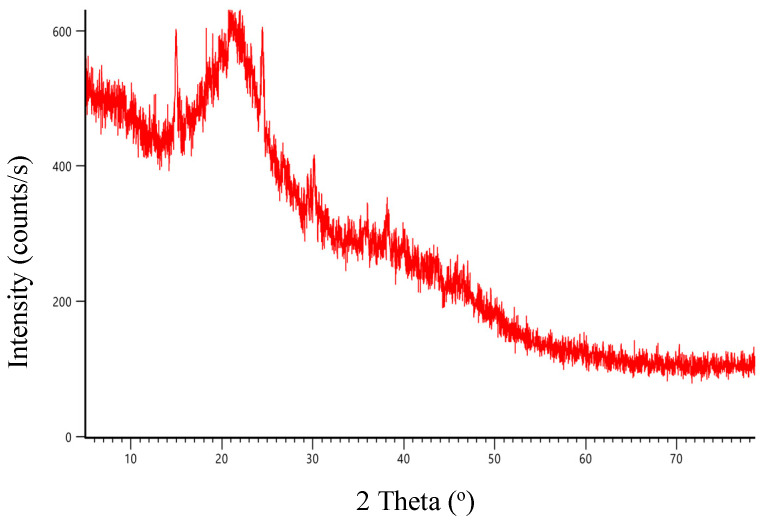
The mulberry leaves’ XRD pattern.

The XRD graph demonstrates a strong correlation between the structure depicted in Figure 4 and the powder data for mulberry leaves subjected to compression. As illustrated in the spectrum, the 2 Theta (°) values are 14.93°, 18.97°, 20.80°, 24.45°, 30.14°, 38.19°, and 86.48°. The broad peaks observed indicate that the adsorbent structure shows amorphous properties.

#### 3.1.4. BET Analysis

**Figure 5:**
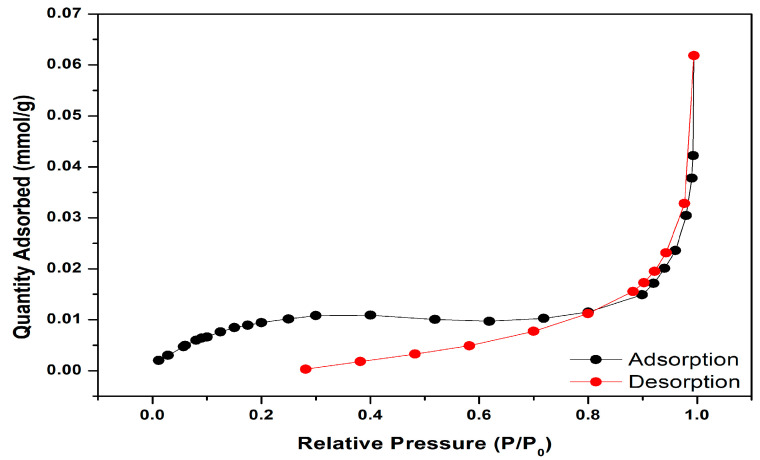
The mulberry leaves’ BET pattern.

Figure 5 presents the nitrogen adsorption–desorption isotherms of mulberry leaves, exhibiting a Type IV hysteresis loop characteristic of mesoporous materials. BET analysis quantified the adsorbent’s textural properties, revealing a surface area of 0.96 m^2^/g, a pore volume of 0.002 cm^3^/g, and an average pore width of 8.99 nm. Based on IUPAC pore-size classification, where macroporous (>50 nm), mesoporous (2–50 nm), microporous (0.7–2 nm), and ultra-microporous (<0.7 nm) materials are distinguished [73,74]. The measured pore width of 8.96 nm confirms the mesoporous structure of the mulberry leaf adsorbent.

As evidenced by the data obtained from XRD and BET measurements, it is understood that mulberry leaves with amorphous and mesoporous adsorbent properties exhibit results compatible with the SEM images in Figure 2.

### 3.2. Determination of pH Point of Zero Charge

The pH point of zero charge (pH_pzc_) represents the pH at which the adsorbent’s net surface charge equals zero or becomes neutral. pH_pzc_ was determined through the intersection point of the initial pH (pH_i_) and final pH (pH_f_) curves (x-axis) [75]. In the present study, due to its operational simplicity and established reproducibility, the widely adopted drift method was employed to measure the pH_pzc_ value of the mulberry leaf adsorbent [76]. The pH of mulberry leaves was measured to identify whether they were acidic or basic. To determine the pH_pzc_ value of the mulberry leaf adsorbent, 40 mL of a 0.1 M NaNO_3_ solution was added to a 100 mL glass beaker (11 pieces in total), and the drift method was employed in this study. Afterward, each solution’s initial pH (pH_i_) was systematically adjusted with a pH range of 2 to 12 using 0.1 M HCl or 0.1 M NaOH solutions. Subsequently, 0.5 g of adsorbent was added to each glass beaker, and the mixed solutions were equilibrated at laboratory room temperature with continuous agitation for 48 h. Then, a pH meter was used to determine the final pH of each solution (pH_f_). A graph is created with the x-axis representing the initial pH of the solution (pHi) and the y-axis representing the ΔpH values (ΔpH = pH_f_ − pH_i_).

**Figure 6:**
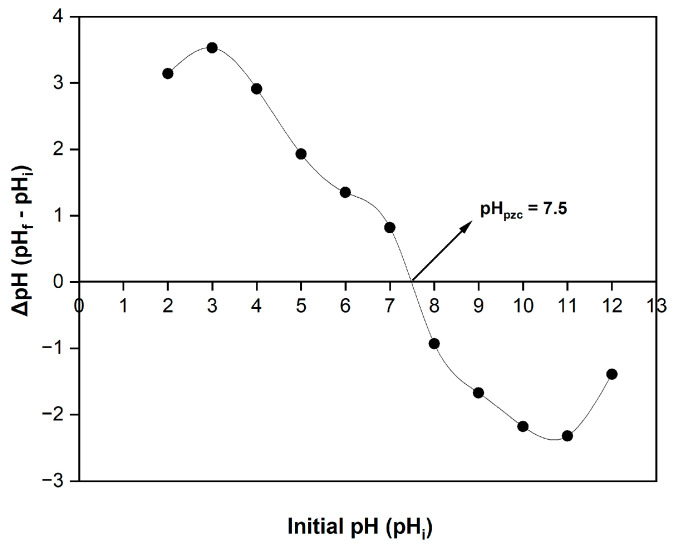
Point of zero charge (pH_pzc_) for mulberry leaf adsorbent.

As shown in Figure 6, the pH_PZC_ value of the mulberry leaf adsorbent used is 7.5. It is known that at pH levels below the pH_PZC_, the positive charges on the adsorbent surface will increase. Increasing H^+^ content will lead to an increase in the repulsive force between the adsorbent and the cationic dye molecules. Therefore, a decrease in cationic dye removal efficiency is expected. As seen in the later sections of the study, the % removal decreases at pH < pH_PZC_. Conversely, increasing the negative charge around or above the pH_PZC_ value will increase the cationic dye removal. Experimental data support the finding that cationic dye removal increases at higher pH values in the later sections of the study.

### 3.3. Effect of Initial Dye Concentration on Adsorption of BY28 and BB3

All optimization studies were conducted by calculating the mean and standard deviation at a confidence interval of 95%, with three replicates for each variable. The investigation examined the influence of initial dye concentration on adsorption efficiency using a specified concentration (5–50 mg/L) for both BY28 and BB3 dyes. The natural pH value of these mixtures is around pH 6.1 for BB3 and 6.5 for BY28. For each experiment, 50 mL of BY28 and BB3 dye solutions at specific concentrations were taken in a 100 mL glass beaker, and 0.5 g of mulberry leaves as an adsorbent was weighed and added. The mixtures were agitated at 150 rpm for 60 min using a temperature-controlled water bath shaker maintained at ambient laboratory conditions (25 ± 2 °C). The mixed solution was filtered to separate the solid and liquid phases using filter paper (Whatman Cat Number 1001-150).

**Figure 7:**
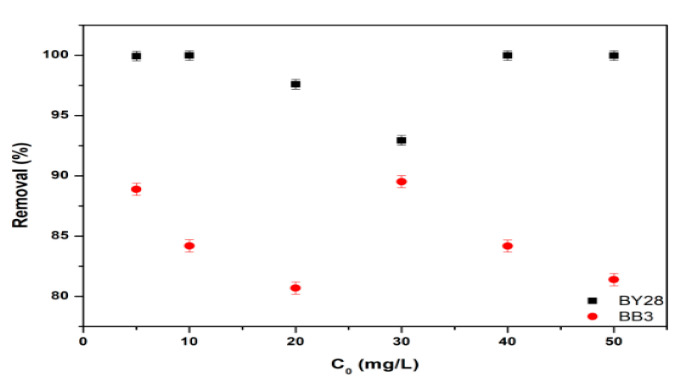
Effect of initial dye concentration on the removal efficiency of BY28 and BB3.

**Figure 8:**
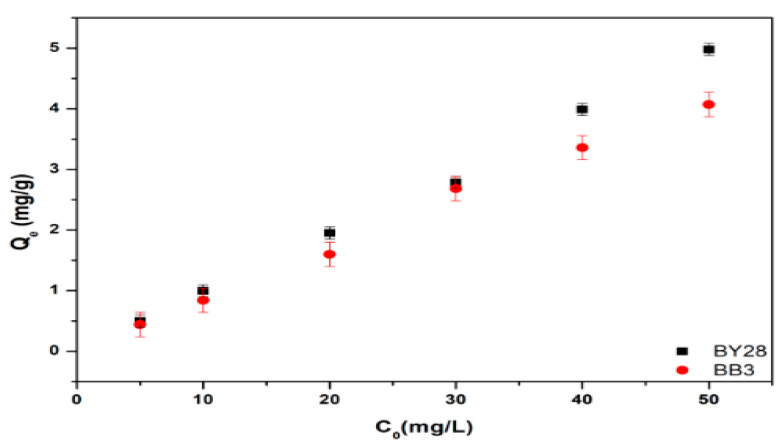
Effect of initial dye concentration on the adsorption capacity of BY28 and BB3.

Figure 7 and Figure 8 show the results of the optimum values of the removal efficiency and adsorption capacity. They show that the optimum concentrations for the percentage removal (%R) of BY28 and BB3 dyes are 10 mg/L and 30 mg/L, respectively.

At the optimal BY28 dye concentration of 10 mg/L, the removal efficiency reached 99.99%. However, at a concentration of 5 mg/L of BY28 dye, there is still a lot of free space in which the dye has not been filled; thus, it has not reached optimal conditions. In contrast, the removal percentage of BB3 dye at 30 mg/L increased to 89.5%, suggesting that the available adsorption sites were effectively occupied, with the functional groups of the mulberry leaf adsorbent forming bonds with the dye molecules. However, at higher concentrations (40 and 50 mg/L), the adsorption efficiency declined to 84.2% and 81.4%, respectively. This reduction can be attributed to the disproportionate ratio of dye molecules to available adsorbent sites, leading to saturation and diminished removal performance. Additionally, the different molecular weights and solubility values of the two dyes may result in varying % removal rates. (Their solubility in water at 20 °C is BB3 = 40 g/L, BY28 = 8.8 g/L.)

### 3.4. The Effect of Varying Amounts of Adsorbent on the Adsorption of BY28 and BB3

The influence of adsorbent dosage on the removal of BY28 and BB3 dyes was investigated using varying quantities (0.1, 0.3, 0.7, 0.9, and 1.5 g). Precisely measured amounts of the adsorbent were introduced into five separate glass beakers. Each beaker was then filled with 50 mL of dye solution, prepared at concentrations of 10 mg/L for BY28 and 30 mg/L for BB3. The mixtures were agitated at a constant shaking speed of 150 rpm and maintained at a temperature of 25.0 ± 0.2 °C for 60 min to ensure uniform interaction between the adsorbent and dye molecules.

**Figure 9:**
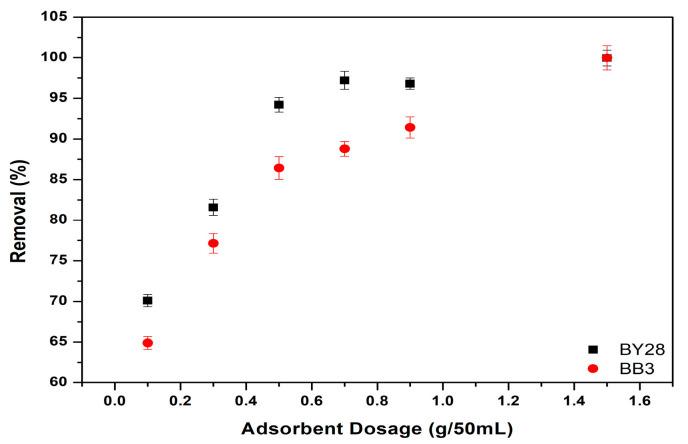
Effect of adsorbent amount on removal efficiency of BY28 and BB3.

**Figure 10:**
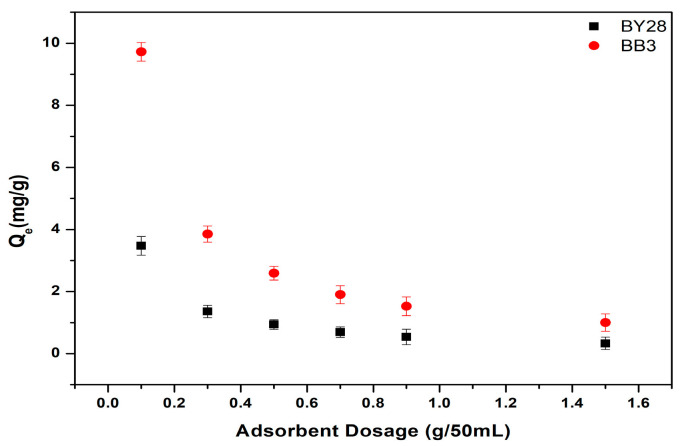
Effect of adsorbent amount on adsorption capacity of BY28 and BB3.

The results of the optimum values of the removal efficiency and adsorption capacity are shown below in Figure 9 and Figure 10. It is shown that the optimum values for the percentage removal (%R) of BY28 and BB3 dyes are both found when the adsorbent dosage is 1.5 g per 50 mL. The results demonstrate a direct correlation between adsorbent dosage and removal efficiency, with higher dosages yielding improved dye elimination. This enhancement in adsorption performance for both dyes can be ascribed to the expanded surface area and greater availability of active binding sites provided by increased adsorbent quantities.

### 3.5. Effect of Contact Time on Adsorption of BY28 and BB3

The influence of contact time on BY28 and BB3 dye adsorption was investigated using 50 mL of 10 mg/L (BY28) and 30 mg/L (BB3). These dye solutions were transferred to separate 100 mL glass beakers. Afterward, the predetermined optimal adsorbent amount of 1.5 g was weighed and added. The mixtures were agitated at 150 rpm while maintaining a constant temperature of 25.0 ± 0.2 °C across varying time intervals ranging from 5 to 180 min.

**Figure 11:**
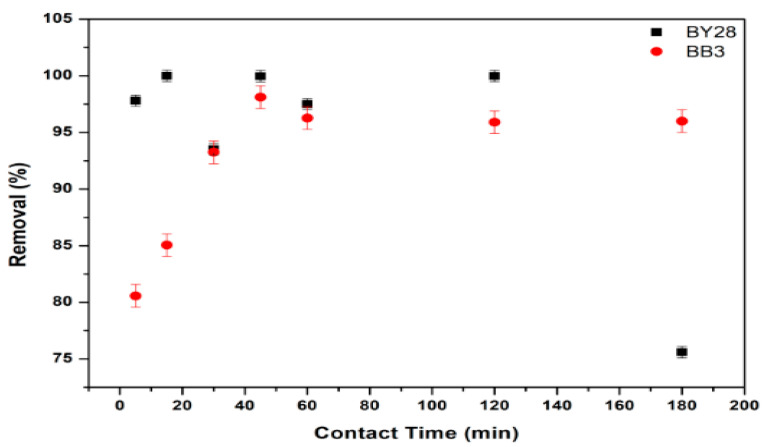
Effect of contact time on removal efficiency of BY28 and BB3.

The optimum contact time is the minimum time required by the mulberry leaf adsorbent during the process of maximum adsorption of BY28 and BB3 dyes (adsorbate) until it achieves its saturated condition, as shown by the high percentage of removal (%R). The experimental results demonstrate that once an adsorption equilibrium is attained, extended contact duration between the adsorbent and adsorbate exhibits minimal influence on the overall dye adsorption kinetics. In a contact time experiment, the optimum removal efficiency values are seen in Figure 11. This graph shows that the percentage removal (%R) of BY28 and BB3 dyes is optimal for 15 and 45 min contact times, respectively.

Figure 11 indicates that the adsorption of BY28 dye adsorbed by the adsorbent at 15 min has the best percentage removal of 99.99%. This shows that the empty spaces of the adsorbent have been filled by the adsorbate (dye) so that an equilibrium is obtained. The adsorption efficiency of BY28 dye exhibited a marked decline at the maximum contact duration of 180 min, yielding substantially reduced removal percentages. This is due to the longer contact time between the adsorbent and adsorbate, which allows for the desorption and detachment of the dye that the adsorbent has adsorbed. After 45 min of contact time, the adsorption of BB3 dye reached equilibrium between the adsorbent and the adsorbate (dye), resulting in optimal adsorption, with a removal percentage of 98.12%.

### 3.6. Effect of Temperature on Adsorption of BY28 and BB3

**Figure 12:**
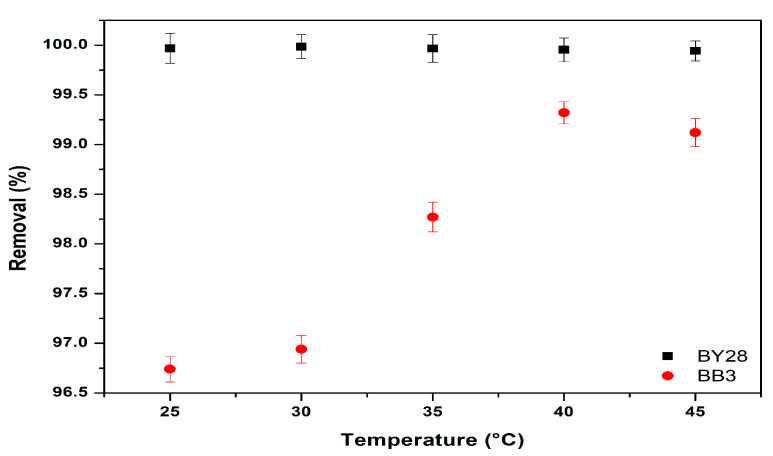
Effect of temperature on the removal efficiency of BY28 and BB3.

To examine the effect of temperature on BY28 and BB3 dye solutions, experiments were conducted under controlled conditions: 50 mL of each dye solution at their respective optimal concentrations (10 mg/L for BY28 and 30 mg/L for BB3), a stirring speed of 150 rpm, specified contact times (15 min for BY28 and 45 min for BB3), and an adsorbent dosage of 1.5 g across a temperature range of 25 to 40 °C. The optimal removal efficiencies, as depicted in Figure 12, demonstrate that the highest dye removal percentages (%R) were achieved at 30 ± 0.2 °C for BY28 and 40 ± 0.2 °C for BB3. At equilibrium, the removal efficiency of BB3 increased from 96.7% to 99.3% as the temperature rose from 25 to 40 °C. In contrast, the removal efficiency of BY28 exhibited a slight increase from 99.97% to 99.99% when the temperature was elevated from 25 to 30 °C.

### 3.7. Effect of Stirring Speed on Adsorption of BY28 and BB3

The influence of stirring speed on the adsorption of BY28 and BB3 dye solutions was investigated. For the BY28 dye, 50 mL of a 10 mg/L solution was put into a 100 mL glass beaker. Then, 1.5 g of an adsorbent was added. The mixture was agitated at 30 °C at varying speeds (100–180 rpm) for 15 min before filtering through filter paper.

A parallel procedure was conducted for the BB3 dye under its predetermined optimal conditions: 50 mL of a 30 mg/L solution, 1.5 g of adsorbent, a temperature of 40 °C, and a contact time of 45 min.

**Figure 13:**
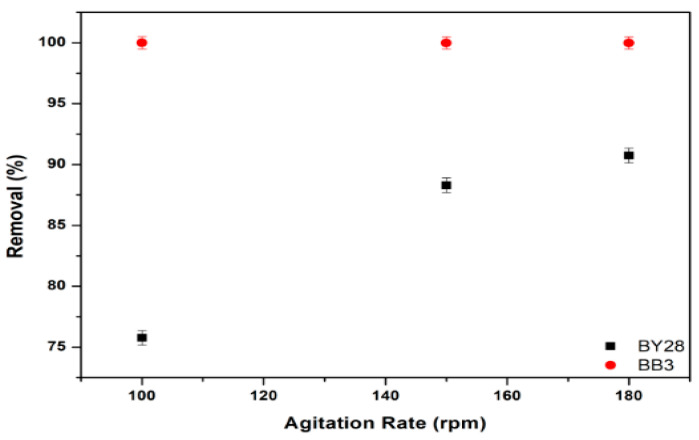
Effect of shaking speed on the removal efficiency of BY28 and BB3.

The optimum values of removal efficiency are shown in Figure 13. The results indicate that the percentage removal (%R) of BY28 and BB3 dyes is optimal at stirring speeds of 180 rpm and 100 rpm, respectively.

With increasing agitation rates, the percentage removal of the BY28 dye rose from 75.8% to 90.7%. An increasing agitation rate may lower the film boundary layer by enclosing the adsorbent particles, enhancing the rate of external film diffusion and adsorption. Nevertheless, the agitation rate does not influence the rate of BB3 dye adsorption on mulberry leaf adsorbent. This can be attributed to the rapid adsorption of the BB3 dye by mulberry leaves. It can be established that the influence of agitation is contingent upon the specific characteristics of both the adsorbent and the adsorbate.

### 3.8. Effect of pH on Adsorption of BY28 and BB3

During adsorption studies, basic parameters such as initial dye concentration, adsorbent dose, contact time, ambient temperature, and agitation speed were evaluated. In addition, pH value is one of the basic parameters affecting the result. When the pH value of the solution changes, a difference in the absorbance value is expected, as the % removal amount will change. To assess the effect of pH on the adsorption of BY28 and BB3 dyes, 50 mL of a 10 mg/L BY28 solution was added to a 100 mL glass beaker. Subsequently, 1.5 g of adsorbent was weighed and added. The pH was adjusted across a range of 4 to 10 in separate beakers, and the mixtures were agitated at 180 rpm at 30 °C for 15 min.

An identical procedure was applied to the BB3 dye solution. A 50 mL measure of a 30 mg/L BB3 solution was transferred into a 100 mL glass beaker, and 1.5 g of adsorbent was weighed and added. A pH solution ranging from 4 to 10 was added to each glass beaker. The mixture was then agitated at 100 rpm and maintained at 40 °C for 45 min.

**Figure 14:**
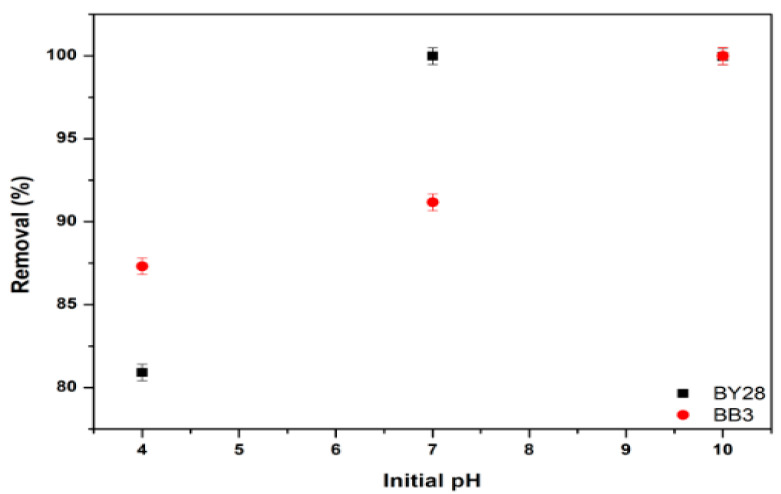
Effect of pH on the removal efficiency of BY28 and BB3.

As shown in Figure 14, the optimum values of the removal efficiency were obtained. It is demonstrated that the percentage removal (%R) of BY28 is optimum in neutral conditions at pH 7. Meanwhile, the BB3 dye solution is optimal in an alkaline environment at a pH 10.

During adsorption at pH 4, the high concentration of H^+^ charge on the surface of the adsorbent increases the repulsive forces between the cationic dye and the adsorbent. This causes a decrease in the amount of dye molecules bound to the adsorbent and removed from the environment and therefore in the % removal value [77]. Furthermore, since pH 4 < pH_pzc_ 7.5, the increased proton mobility caused by the H+ ion increases the repulsive effect between the adsorbent biomass and the dye, resulting in extremely low % removal values. Conversely, as the pH approaches and exceeds the pH_pzc_ value, a more negative charge density will develop, increasing the % removal value.

Similarly, considering the molecular structures of dye compounds, it is anticipated that their pKa values may be high due to the functional groups they contain (quaternary amine, hydroxyl (–OH), amine, etc.). In this case, when the pH value is lower than the pKa value (pH 4), the low % removal resulting from poor adsorption is confirmed by the increased % removal when the pH value is higher (pH 7, 10).

The results from the experiments to determine the optimal conditions are shown in Table 1. All optimization tests were performed using a standard water bath shaker.

### 3.9. Ultrasonic Water Bath Shaker Experiment

To investigate the effect of the ultrasonic water bath shaker on the adsorption process of BY28 and BB3 dye solutions, experiments were repeated in an ultrasonic water bath under optimal operating conditions. For this purpose, 50 mL of a 10 mg/L BY28 solution was placed in a 100 mL glass beaker, and 1.5 g of adsorbent was weighed and added. The dye solution was shaken at 30 °C for 15 min. The stirred solution was filtered using filter paper.

The same procedure was repeated for the BB3 dye solution: 50 mL of a 30 mg/L BB3 solution was added to a 100 mL glass beaker, and 1.5 g of adsorbent was weighed and added. The dye solution was shaken at 40 °C for 45 min. Filtration of the stirred solution was carried out using filter paper.

**Figure 15:**
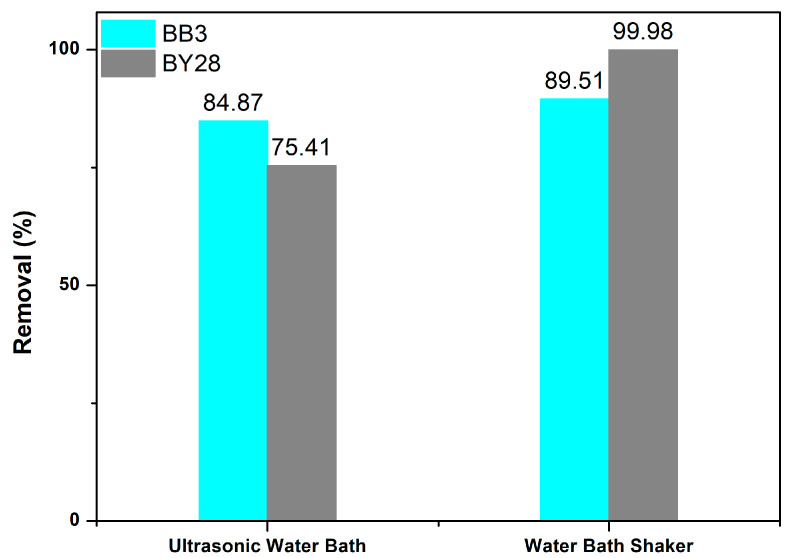
Comparison of removal efficiency results between normal and ultrasonic water bath shakers.

Figure 15 illustrates the optimal removal efficiency of both dyes when using ultrasonic water and bath shakers. It indicates that the percentage removal (%R) of BY28 and BB3 dyes is better when using a standard water bath shaker in optimum conditions (for BY28 10 mg/L; 1.5 g; 15 min; 30 °C and for BB3 30 mg/L; 1.5 g; 45 min; 40 °C), which can reach more than 99%.

Under optimal conditions, ultrasound-assisted experimental studies were conducted at 120 W and 40 kHz. As seen in Figure 15, there is a slight decrease in the percentage removal values of the studied dyes. The components in the structure of the adsorbent used may have interacted with the solution medium in the ultrasonic environment and caused a negative effect on the results. The decrease in removal values observed in studies using ultrasonic baths is likely caused by structural changes from cavitation effects generated by ultrasonic waves [78]. These effects can weaken the dye–adsorbent interaction and also lead to desorption. Nevertheless, it provides significant results with removal rates of 75.41% for BY28 and 84.87% for BB3.

### 3.10. Use of Real Water Sources Experiments

Previous experiments were conducted under laboratory conditions, utilizing distilled water exclusively during adsorption and dilution processes. In this real-sample experiment, the adsorption of BY28 and BB3 dye solutions was examined using three different types of water: Kurtmekan River water, running tap water, and distilled water. The objective was to investigate whether other types of water affect the effectiveness of dye solution removal when an adsorption method uses mulberry leaves as an adsorbent.

A stock solution was prepared by dissolving 0.001 g of BY28 and 0.003 g of BB3 in separate 100 mL volumetric flasks to achieve final concentrations of 10 mg/L and 30 mg/L, respectively. The flasks were filled to the mark with three types of prepared water: water from the Kurtmekan River, running tap water, and distilled water.

To investigate the adsorption of BY28, 50 mL of a 10 mg/L dye solution was added to a 100 mL glass beaker. Subsequently, 1.5 g of the adsorbent material was precisely weighed and added to the solution. The mixture was agitated at 180 rpm and maintained at 30 °C for 15 min. Following adsorption, the solution was filtered through filter paper to separate the adsorbent.

The same experimental procedure was employed for the BB3 dye solution: 50 mL of a 30 mg/L BB3 solution was transferred into a 100 mL glass beaker, and 1.5 g of adsorbent was added. It was adjusted to pH 10 by introducing an alkaline solution. The dye solution was stirred at 100 rpm at 40 °C for 45 min. 

To evaluate the experimental results, standard measurement curves were generated using different water sources, as shown in Table 2. As indicated by the equations, the slopes for both dyes follow a clear order of river water > tap water > distilled water. This is probably due to the different ionic strengths in the water sources.

**Figure 16:**
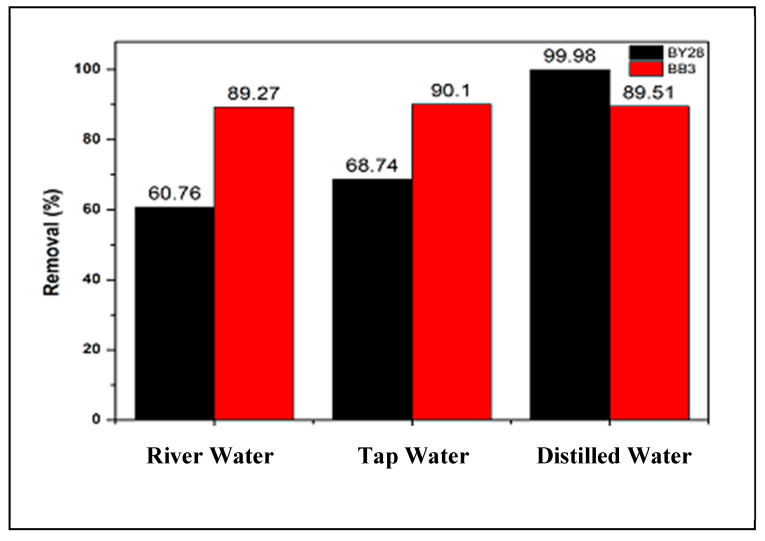
Real sample test results.

As seen in Figure 16, the results of a real-sample experiment with the optimum removal efficiency values were obtained. This graph illustrates the effect of changing water type on the %R of BY28 and BB3 dye solutions.

As shown in Figure 16, in studies involving BY28, removal rates of 60.76%, 68.74%, and 99.98% were observed when river water, tap water, and distilled water were used, respectively. Resources other than distilled water exhibited a slight decrease in removal rates due to the presence of different components. Despite this, a significant amount of BY28 dye was still removed. Similarly, when BB3 dye was used with river water, tap water, and distilled water at 89.27%, 90.1% and 89.51%, respectively, the decrease in the observed percentage removal values may be due to the presence of cationic components that may be present in river and tap water solutions. The values found show that dyes can be removed to a certain percentage in natural water resources.

### 3.11. Regeneration and Reusability of Adsorbent

The regeneration and reusability of mulberry leaves as adsorbents were investigated using a batch method to determine their efficacy in repeated adsorption of BY28 and BB3 dyes from wastewater. Experiments were conducted on both dyes separately to observe the best desorption agent from three variations of solutions, including 0.1 M HCl, 0.1 M NaOH, and 0.1 M CH_3_COOH, for 10–15 min, and agitated at 150 rpm to regenerate the surface of the mulberry leaves containing dyes. Before reuse, the adsorbent was rinsed three times with ultrapure water and subsequently oven-dried at 65 °C for 24 h to achieve a constant mass. The regenerated mulberry leaf adsorbent was reused in the next BY28 and BB3 adsorption tests for up to two repetitions.

The best desorption agent provides the most significant percentage of desorption or removal, while the best adsorbent regeneration agent provides the highest adsorption capacity upon reuse. This study focused on the desorption agent that best regenerates the mulberry leaf adsorbent.

In this study, the adsorption process was carried out with two reuses of mulberry leaf adsorbent. The volume of the solution containing both dyes used in the initial adsorption was 50 mL, and 1.5 g of adsorbent was used under optimum conditions. During the reuse process, the quantity of the dye solution was adjusted to correspond with the residual adsorbent mass while maintaining the initial adsorption proportion of both dyes.

The desorption process was applied to mulberry leaves used in the adsorption of both dyes under optimal conditions, including concentration, adsorbent dosage, agitation speed, and time. The experiment was repeated three times in the adsorption process, which consists of adsorption I (initial), adsorption II (reuse I) after desorption I, and adsorption III (reuse II) after desorption II. The optimum conditions for parameter variations in this study are shown in Table 1.

**Figure 17:**
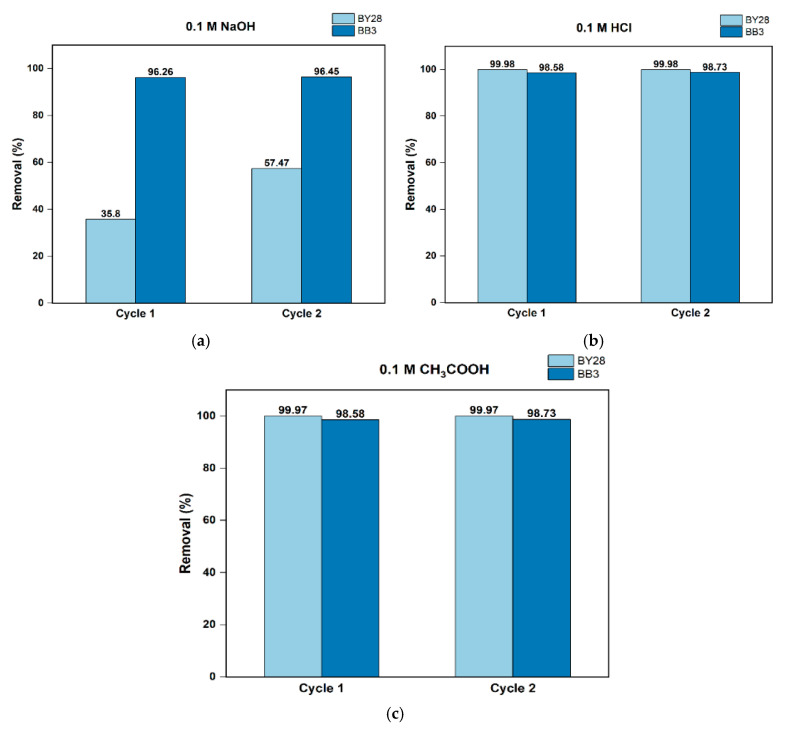
The reusability performance of an adsorbent through the two-cycling of adsorption or desorption of BY28 and BB3 dyes: (**a**) 0.1 M NaOH, (**b**) 0.1 M HCl, and (**c**) 0.1 M CH_3_COOH.

The results obtained from the regeneration experiments are shown in Figure 17. Even after two iterations, the adsorption capacity of the mulberry leaf yielded quite good results. As shown in Figure 17a, the highest removal value in the regeneration performed with 0.1 M NaOH was 96.45% for BB3. The increase in the adsorption capacity observed between the first and second regeneration steps, suggests that the NaOH solution, may have caused the removal of any blockages in the adsorbent surface pores or changes in the ionization states of functional groups that may have a positive impact on the % removal value. Similarly, the 99.98% removal value for BY28 using 0.1 M HCl is shown in Figure 17b. In the removal studies performed using 0.1 M CH_3_COOH solution in Figure 17c, the highest removal value of 99.97% was achieved in BY28.

The experimental findings showed that acidic desorption agent solutions significantly improved both dyes’ removal percentage and reusability potential. When immersed in an acidic medium, the adsorbent’s carboxyl, carbonyl, or hydroxyl functional group undergoes a protonation process, thereby losing its capacity to bind positively charged ions. This results in the release of ions into the solution or desorption agent. Furthermore, protons (H^+^) in the aqueous phase displace adsorbed ions on the adsorbent surface. The H^+^ ions released by proton source agents such as HCl and CH_3_COOH strongly bind to the adsorbent surface. The low percentage of removal obtained with the basic agent (0.1 M NaOH) is probably related to the fact that the groups become less protonated, making it difficult to detach the bound ions from the adsorbent. Moreover, regeneration with bases also causes a lower percentage of removal because bases do not cause protonation.

Furthermore, BY28 and BB3 probably have different pKa values due to the influence of various functional groups in their structures. The regeneration performance relies on the interaction between the dye’s pKa and the pH of the regeneration solution. Therefore, even when using the same regenerant, different dyes can exhibit different regeneration efficiencies.

### 3.12. Application of Adsorption Isotherms 

**Figure 18:**
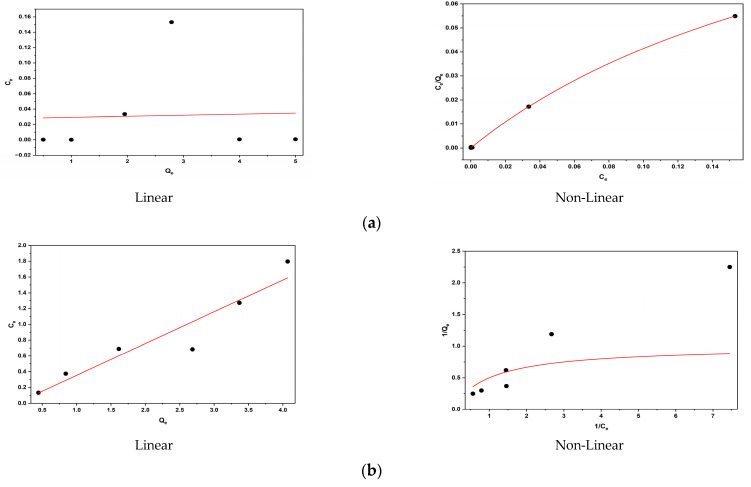
The isotherm plots of the linear and non-linear Langmuir for the adsorption of BY28 (**a**) and BB3 (**b**) dyes using mulberry leaves as an adsorbent.

**Figure 19:**
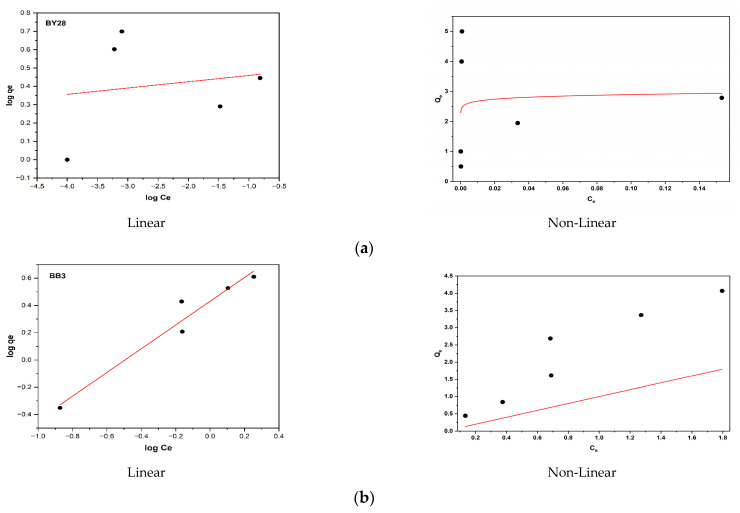
The isotherm plots of the linear and non-linear Freundlich equations for the adsorption of BY28 (**a**) and BB3 (**b**) dyes using mulberry leaves as an adsorbent.

The linear Freundlich and non-linear Langmuir adsorption isotherm models have been plotted in Figure 18a,b and Figure 19a,b, respectively. The Langmuir isotherm constants, particularly the adsorption capacity (q_e_) expressed in mg/g, were derived from the non-linear regression analysis, utilizing the plots of C_e_/q_e_ versus C_e_ (Equation (6)) for BY28 dye and 1/q_e_ versus 1/C_e_ (Equation (5)) for BB3 dye. Meanwhile, the Freundlich isotherm constants, K_f_ (L/mg) and 1/n, were determined from the linear regression of log(q_e_) versus log(C_e_). The calculated Langmuir and Freundlich isotherm parameters for the adsorption of BY28 and BB3 dyes onto mulberry leaves, along with a comparative analysis of their corresponding R^2^ values, are summarized in Table 3. The R^2^ value was used to determine the most appropriate isothermal adsorption model for the adsorption of BY28 and BB3 dyes onto mulberry leaves. The fit of each model is decided by selecting the model with the closest R^2^ value to 1.

The experimental findings demonstrate that the non-linear Langmuir isotherm model exhibits superior fit for both the Basic Yellow 28 (BY28; R^2^ = 0.999) and the Basic Blue 3 (BB3; R^2^ = 0.973) dyes, in contrast to the Freundlich isotherm, which produced lower coefficients of determination (R^2^ = 0.027 for BY28 and R^2^ = 0.951 for BB3). This indicates that the active sides are homogeneously dispersed on the mulberry leaves, where BY28 and BB3 dyes form a monolayer coverage on the adsorbent surface. In addition, the difference in capacity values seen in Figure 10 may be because the optimum operating parameters were obtained by taking the % removal values as a reference and therefore could not fully reflect the maximum capacity of the adsorbent.

### 3.13. Comparative Research of Adsorption Capacity for BY28 and BB3

A comparative analysis of the maximum adsorption capacities of mulberry leaves for the removal of BY28 and BB3 dyes alongside various other adsorbents is summarized in Table 4. Mulberry leaves have a reasonably excellent adsorption potential for eliminating BY28 and BB3 dyes in wastewater. Accordingly, it is anticipated that mulberry leaves may yield promising results in the removal of BY28 and BB3 dyes from wastewater. It is noteworthy that they have a higher potential to remove BY28 dye than some data in the literature [79]. Among the advantages of mulberry leaves are their abundance, renewable nature, and environmentally friendly nature as an adsorbent. These properties suggest that they may yield successful results in the removal of toxic dyes from wastewater.

## 4. Conclusions

Based on experimental findings, the adsorption method using mulberry leaves as an adsorbent effectively removed synthetic dye solutions, especially BY28 and BB3 dyes. The dye removal efficiency exceeded 99% under optimal conditions. The experiments determined the ideal parameters as follows: 50 mL, 10 mg/L, 1.5 g, 15 min, 30 °C, and 180 rpm for Basic Yellow 28 (BY28) at a neutral pH (7) and 50 mL, 30 mg/L, 1.5 g, 45 min, 40 °C, 100 rpm for Basic Blue 3 (BB3) at a basic pH (10). Results showed a positive relationship between adsorbent dosage and removal efficiency. In the experiments carried out with an ultrasonic bath, removal efficiency of 75.41% for BY28 and 84.87% for BB3 was obtained, indicating the supportive effect of the ultrasonic bath on dye removal. Mulberry leaves demonstrated high removal potential for BY28 and BB3 dyes in real-sample applications using both distilled and tap water, with maximum removal percentages of 99.98% for BY28 and 90.1% for BB3. Additionally, the adsorbent regenerated through two cycles with solutions including 0.1 M NaOH, 0.1 M HCl, and 0.1 M CH_3_COOH, remained effectively reusable for removing BY28 and BB3 dyes. The optimal removal efficiencies reached 99.98% for BY28 in 0.1 M HCl and 98.73% for BB3 in 0.1 M CH_3_COOH. Adsorption isotherm analysis revealed that the non-linear Langmuir model provided the best fit, with correlation coefficients (R^2^) of 0.999 for BY28 and 0.973 for BB3. The maximum adsorption capacities were 0.14 mg/g for BY28 and 7.19 mg/g for BB3. Since freshwater is vital for sustaining ecosystems and human life, treating industrial wastewater remains a critical focus of environmental research. It is anticipated that this study can serve as a model for future wastewater treatment using affordable, natural, reusable, eco-friendly, abundant, easy-to-operate, and efficient adsorbents for removing toxic chemicals such as dyes and heavy metals discharged by various industries and human activities.

## Figures and Tables

**Table 1:** Optimum parameter conditions of BY28 and BB3.

	Concentration (mg/L)	Adsorbent Dosage (g)	Agitation Speed (rpm)	Time (min)	Temperature (°C)	pH
BY28	10	1.5	180	15	30	7
BB3	30	1.5	100	45	40	10

**Table 2:** Standard measurement curves were prepared using different water for BY28 and BB3.

BY28–River water	Y = 0.035X + 0.007	R^2^ = 0.997
BY28–Tap water	Y = 0.022X + 0.051	R^2^ = 0.995
BY28–Distilled water	Y = 0.014X + 0.042	R^2^ = 0.999
BB3–River water	Y = 0.042X + 0.071	R^2^ = 0.999
BB3–Tap water	Y = 0.031X + 0.026	R^2^ = 0.998
BB3–Distilled water	Y = 0.023X + 0.056	R^2^ = 0.997

**Table 3:** Langmuir and Freundlich isotherm constants for the adsorption of BY28 and BB3 on mulberry leaves.

	Langmuir Isotherm			Freundlich Isotherm		
	*K_L_* (mg/L)	*q_max_* (mg/g)	*R* ^2^	*K_f_* (mg/L)	1/*n*	*R* ^2^
Linear Isotherm						
BY28	0.050	714.29	0.002	3.118	0.034	0.027
BB3	−8.235	2.483	0.903	2.696	0.871	0.951
Non-linear Isotherm						
BY28	4.099	0.142	0.999	3.131	0.034	0.028
BB3	0.062	7.192	0.973	1	1	−0.422

**Table 4:** Table of literature data for the removal of BY28 and BB3 dyes using different adsorbent materials.

Adsorbent	Adsorption Capacities (mg g^−1^)		Adsorption Model	References
	BY28	BB3		
Mulberry leaves (*Morus nigra* L.)	0.14	10.30	Langmuir	Present study
Ethiopian kaolinite clay	5.71	-	Langmuir	[60]
Macadamia seed husks	-	1.40	Langmuir–Freundlich	[80]
Silybum marianum (SLM) Stem-Natural	36.54	13.96	Langmuir	[81]
Raw cedar sawdust	-	47.62	Langmuir	[82]
Mussel shells	93.45	-	Freundlich	[79]
Polyaniline/Magnetite (Fe_3_O_4_) Composites	-	78.13	Langmuir	[83]
Quarternized sugarcane bagasse	-	37.59	Freundlich	[84]
Activated carbon	769.23	-	Langmuir	[85]
Natural Durian Peel	-	49.50	Langmuir–Freundlich	[86]

## Data Availability

The original contributions presented in this study are included in the article. Further inquiries can be directed to the corresponding author.
